# Adsorption and Sustained Delivery of Small Molecules from Nanosilicate Hydrogel Composites

**DOI:** 10.3390/ph15010056

**Published:** 2022-01-01

**Authors:** Samuel Stealey, Mariam Khachani, Silviya Petrova Zustiak

**Affiliations:** Biomedical Engineering Program, Parks College of Engineering, Saint Louis University, Saint Louis, MO 63103, USA; samuel.stealey@slu.edu (S.S.); mariam.khachani@slu.edu (M.K.)

**Keywords:** laponite, polyethylene glycol hydrogel, acridine orange, therapeutic delivery, nanocomposite, intercalation

## Abstract

Two-dimensional nanosilicate particles (NS) have shown promise for the prolonged release of small-molecule therapeutics while minimizing burst release. When incorporated in a hydrogel, the high surface area and charge of NS enable electrostatic adsorption and/or intercalation of therapeutics, providing a lever to localize and control release. However, little is known about the physio-chemical interplay between the hydrogel, NS, and encapsulated small molecules. Here, we fabricated polyethylene glycol (PEG)-NS hydrogels for the release of model small molecules such as acridine orange (AO). We then elucidated the effect of NS concentration, NS/AO incubation time, and the ability of NS to freely associate with AO on hydrogel properties and AO release profiles. Overall, NS incorporation increased the hydrogel stiffness and decreased swelling and mesh size. When individual NS particles were embedded within the hydrogel, a 70-fold decrease in AO release was observed compared to PEG-only hydrogels, due to adsorption of AO onto NS surfaces. When NS was pre-incubated and complexed with AO prior to hydrogel encapsulation, a >9000-fold decrease in AO release was observed due to intercalation of AO between NS layers. Similar results were observed for other small molecules. Our results show the potential for use of these nanocomposite hydrogels for the tunable, long-term release of small molecules.

## 1. Introduction

Polymeric hydrogels have commonly been used as delivery devices because of their favorable biocompatibility, tunability, and degradation properties, as well as their ability to preserve the bioactivity of encapsulated cargo [1]. The nanoporous mesh structure of polymeric hydrogels lends itself to the slowed release of proteins and other larger therapeutics [2]. Release is typically controlled by diffusion, which is affected by crosslinking structure and density, as well as polymer degradation [3]. However, these hydrogels are susceptible to initial burst release that can lead to unfavorable pharmacokinetics, as well as difficulty in achieving long-term release [4]. These drawbacks are even more prevalent for the release of low-molecular-weight (<1000 Da) small molecules, which can be an order of magnitude smaller than the mesh size of the polymeric hydrogels, leading to high permeability [5]. Due to their small size, these small molecules can rapidly diffuse out of the hydrogel without much hinderance and be completely released into the local environment within a matter of hours [6]. The rapid release kinetics of small molecules limits the utility of traditional polymeric hydrogels for sustained and long-term release applications.

**Figure 1:**
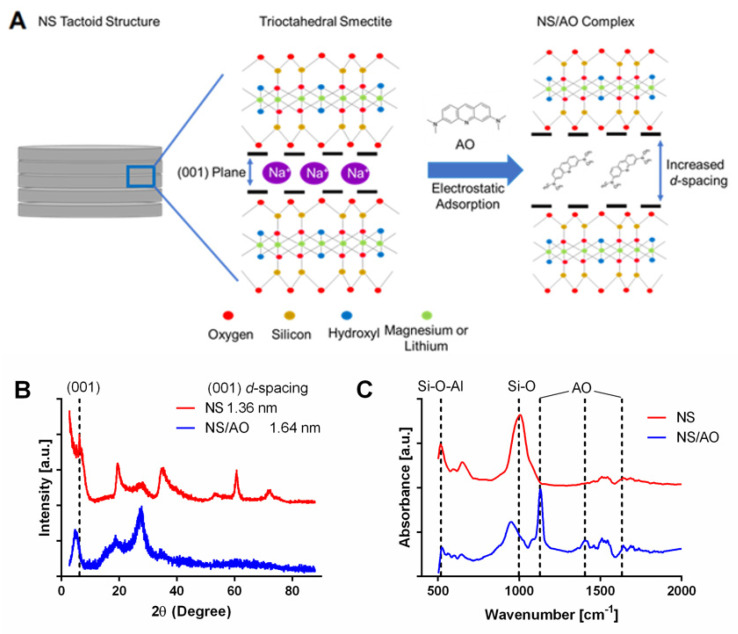
Intercalation of AO between NS particle layers. (**A**) Schematic of intercalation of AO into (001) planes of NS particles, resulting in increased *d*-spacing. (**B**) XRD patterns of NS particles and NS/AO complexes. The leftward shift of the NS/AO 2θ peak at the (001) plane indicates increase in *d*-spacing between NS layers. (**C**) FTIR-ATR spectra of NS particles and NS/AO complexes, indicating interaction with NS silicate groups. Vertical dash lines indicate bands at 533, 991, 1127, 1401, and 1633 cm^−1^.

Several methods have been explored to enhance the retention and prolong the release of small molecules from polymeric hydrogels including tethering of small molecules to the hydrogel structure [7,8], creating stimulus-responsive swelling hydrogels [9], and the incorporation of nanomaterials into the hydrogel matrix [10]. Here, we focus on the use of nanomaterials, specifically two-dimensional layered nanosilicate Laponite XLG (abbreviated hereafter as “NS”), to adsorb small-molecule therapeutics to provide for sustained release. NS particles have been exploited because of their high surface-area-to-volume ratio, unique charge characteristics, biocompatibility, and biodegradability [11,12]. The particles are synthetic disc-shaped trioctahedral smectites with a diameter of 25–30 nm and a height of 1 nm (see Figure 1A) [13,14]. Negatively charged faces and positively charged edges allow for the electrostatic adsorption of charged molecules, and the stacked tactoid structure offers the potential for the intercalation of small molecules in the interlayer space [15]. Importantly, NS are biodegradable and have been shown to not elicit a cytotoxic response [16,17,18]. The incorporation of NS within polymeric hydrogels offers a lever by which release can be controlled, allowing for the tuning of release kinetics from hydrogels.

NS have been exploited for their ability to prolong release of a wide variety of small molecules, ranging from therapeutics to amino acids to peptides [19,20,21]. Small molecules have been shown to adsorb to the surface of NS particles or be intercalated between NS layers [22]. The initial burst release is reduced in the presence of NS, leading to the prolonged release of both small molecules and protein. The bioactivity of adsorbed therapeutics has also been shown to be maintained following interaction with NS [23,24,25]. The NS discs have been used in composite materials such as hydrogels to aid in localized delivery [26]. For example, Cimen et al. developed a gelatin-polyethylene glycol (PEG)/NS hybrid hydrogel to deliver doxorubicin [27].

However, while it has been shown that NS slows small molecule release, effective control of molecule release is harder to achieve because not much is known about the interactions between NS, the hydrogel, and the small molecule as well as the NS-molecule structures that form in a hydrogel. A variety of variables may affect the small molecule release profile, including environmental conditions, polymer–NS interactions, NS–molecule interactions, polymer–molecule interactions, and the associated kinetics, stoichiometry, and concentration-dependency on these variables. NS have been shown to interact with polymeric backbones, including PEG, through secondary crosslinking reactions, which could affect the ability of NS to interact with small molecules [11,28,29]. Functionalized polymers such as hyaluronic acid have also been directly crosslinked with NS [26]. The ability of NS to interact with small molecules could also be affected by the freedom of NS particles to orient themselves in three dimensions, which in turn would be affected by NS incorporation in a hydrogel. These, among many other factors, should be accounted for to develop a plug-and-play type device with a finely tuned release profile. 

Here, we utilize a PEG-NS hydrogel because of PEG’s superb biocompatibility, tunability, bio-inertness, and degradation. The PEG hydrogels used here were formed by Michael-type addition to provide for timed gelation, allowing for observation of the effects of NS on hydrogel gelation kinetics and the corresponding effect on NS–small-molecule interactions. Acridine orange (AO) was used as a model small molecule because of its innate fluorescence, allowing for easy detection. NS/AO interactions were characterized in solution to confirm AO adsorption/intercalation onto NS particles. Next, various nanocomposite hydrogel formulations were explored to elucidate the relationship between PEG, NS, AO, and NS/AO complexes. The conditions explored included varying freedom of NS particles to interact with AO, NS/AO incubation time prior to hydrogel encapsulation, and NS concentration. To confirm the utility of this PEG-based nanocomposite hydrogel as a robust delivery platform, two other model small molecules were used, namely Alexa 647 and Atto Rho13. The developed nanocomposite hydrogels showed great promise as a versatile release device with tunable release characteristics. 

## 2. Results

### 2.1. Characterization of AO Adsorption onto and Intercalation with NS to Form NS/AO Complexes

Most experiments were performed with AO as a model small molecule due to its net-positive change in physiologic pH as well as intrinsic fluorescence, which allowed for easy detection and quantification. Two other model small molecules, Alexa 647 and Atto Rho 13, were used in key experiments to generalize our findings. All molecules used were fluorophores with a molecular weight ≤1000 Da and a net charge of +1 (Table 1) to allow for electrostatic adsorption onto the negatively charged faces of the NS particles. 

We first studied the NS/AO interactions in solution in the absence of a PEG hydrogel to determine the interaction mechanism and the resultant NS/AO complex structure and size. X-ray diffraction was used to examine the effect of AO interaction with NS particles (Figure 1A,B). The diffraction (001) basal reflection plane, which represents the interlayer spacing between NS particles, was shifted towards lower 2θ angles for NS/AO complexes compared to NS only (6.3° to 5.1°). Such a shift represented an increase in interlayer *d*-spacing between NS particles due to AO adsorption and intercalation between NS layers, likely due to an interaction with the silicate SiO_4_^−^ on the faces of NS particles [30]. Furthermore, the majority of peaks found in the NS spectrum showed very little shifting, indicating that the crystalline structure of NS was preserved following NS/AO interactions [31]. 

FTIR-ATR spectra were recorded to confirm the presence of AO in the interlayer region of NS particles. As shown in Figure 1C, a typical peak for NS was observed at 991 cm^−1^ corresponding to Si-O and Si-O-Si stretching band of the silicate layer. In addition, the bands at 472 and 533 cm^−1^ could be assigned to Si-O-Mg and Si-O-Al, respectively. In the NS/AO spectra, an intense and well-resolved peak at 1127 cm^−1^ can be seen, which is characteristic of in-plane β_CH_ of AO, which would indicate that the AO was intercalated into NS layers in its cationic form [30]. Additionally, some characteristic peaks of AO appeared in the NS/AO spectra, especially in the fingerprint region of 1000–1700 cm^−1^, which is indicative of complexation of NS and AO. The peak present at 1633 cm^−1^ could be indicative of the presence of the cationic form of AO (AOH^+^), which was blue-shifted from the neutral form peak that has been reported at 1603 cm^−1^ [22,32] and seen in the AO only spectrum (Supplemental Figure S1). The peak located at 1508 cm^−1^ can be attributed to an aliphatic σ_CN_ stretch coupled to aromatic σ_CC_ stretch motion present in the protonated form of AO [22]. The band at 1401 cm^−1^ is indicative of stretching of σ_CN_, which further indicated the presence of AO within the NS/AO spectrum [32] and was absent in the AO only spectrum. Bands that may be indicative of perpendicular orientation of AO with respect to the faces of NS particles in the (001) plane at 1330 cm^−1^ and 1600 cm^−1^ were not clearly visible here, though others have reported such an orientation of polyheterocyclic compounds in the presence of flat surfaces [22,33]. 

**Figure 2:**
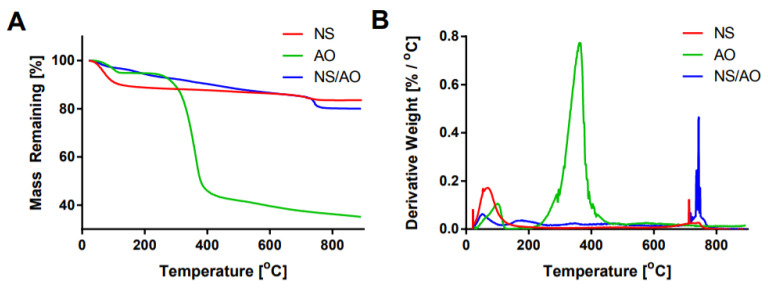
Thermogravimetric analysis of NS, AO, and NS/AO complexes. Percent mass remaining (**A**) and derivative weight (**B**) as a function of temperature for NS only, AO only, and NS/AO complexes reveal interaction between AO and NS particles.

Thermogravimetric analysis (TGA) was performed to confirm the interaction between AO and NS particles (Figure 2). In the NS-only sample, two main regions of mass loss were present: one at ~100 °C and another at ~700 °C, which represent the loss of physically adsorbed water along with hydrogen bonded water within the NS layers and breakdown of structural hydroxyl groups, respectively [34]. The broad endothermic peak recorded at ~730 °C followed by a sharp exothermic peak at ~755 °C demonstrated the de-hydroxylation of NS occurred simultaneously with a recrystallization reaction, as seen by differential scanning calorimetry (Supplemental Figure S2).

AO showed a slight mass loss around 100 °C of around 5%, presumably due to the loss of adsorbed water molecules, and a significant mass loss of 45% at ~350 °C that represents the degradation of the AO molecules [22,32]. NS/AO complexes showed substantially reduced mass loss at 100 °C compared to NS only or AO only, which indicates that NS/AO interaction led to changes in the status of NS. Thus, the lower weight loss suggests that the hydrogen-bonded water within the NS layers was replaced by AO molecules. A relatively consistent mass loss occurred from ~200 °C to ~700 °C, most likely due to the degradation of surface adsorbed AO and intercalated AO within the NS complexes, as NS only showed little mass loss in this region [30]. Significantly, less mass loss occurred within this region in the NS/AO group compared to AO only, which could be attributed to the formation of NS/AO complexes, which shielded AO molecules from degradation. 

**Figure 3:**
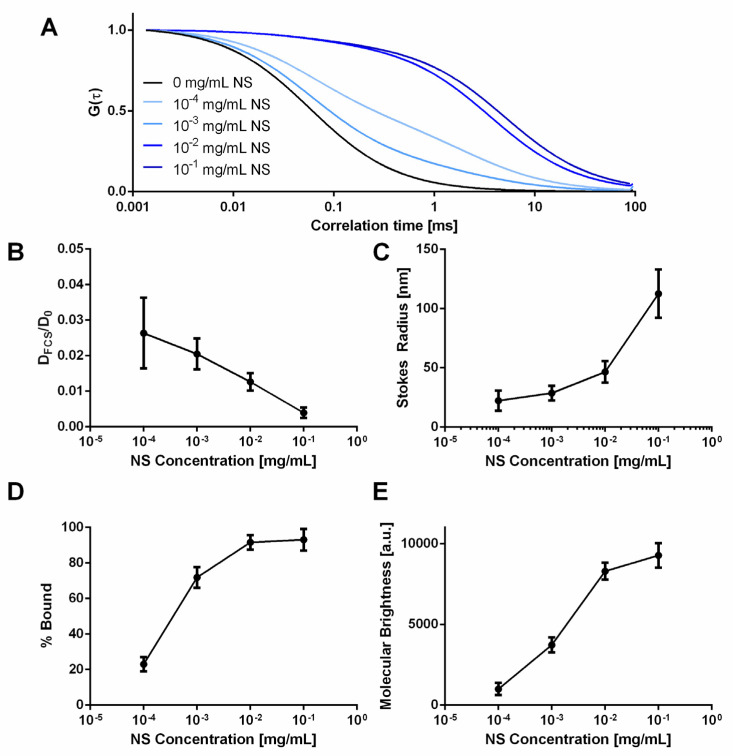
Effect of NS concentration on AO diffusivity, molecular brightness and percent AO bound to NS. (**A**) Normalized autocorrelation functions of AO for various NS concentrations. (**B**) Normalized diffusivities of AO incubated with NS compared to free AO in water. (**C**) Calculated Stokes radius of NS/AO complexes with varying NS concentrations. (**D**) Calculated percentage of AO bound to NS particles as a function of NS concentration. (**E**) Molecular brightness of AO as a function of NS concentration.

To further examine NS/AO interactions, FCS was used to directly measure AO diffusivity in the absence and presence of varying concentrations of NS in solution (Figure 3). At all concentrations of NS, AO diffusivity was significantly decreased, as indicated by the rightward shift of the autocorrelation curves (Figure 3A) and the decrease in normalized diffusion coefficient (Figure 3B), where the diffusion coefficient decreased 33-fold at an NS concentration of 0.0001 mg/mL and 250-fold at an NS concentration of 0.1 mg/mL. Using Equation (5), the radius of the measured NS/AO complex was calculated (Figure 3C). The radius of the NS/AO complexes increased significantly as the NS concentration increased, indicating the formation of larger, tactoid-like complexes due to the intercalation of AO in the NS interlayer space when NS concentration was sufficiently high. At lower NS concentrations, AO adsorption onto NS surfaces may have been predominant. At all NS concentrations tested, the calculated radius was significantly greater (>40-fold increase) than that of AO in the absence of NS. Furthermore, the percent of AO bound to NS increased from 23% at NS of 0.0001 mg/mL to a plateauing value of ~92% at 0.1 mg/mL NS (Figure 3D). A two-component fit (Equation (3)) was used to fit all measurements from solutions containing NS, indicating the presence of two distinct diffusing species: a rapidly diffusing species (free AO) and a slowly diffusing species (large NS/AO complexes). The molecular brightness was also measured as a function of NS concentration, representing the brightness of fluorescent molecules passing through the focal volume (Figure 3E). As the NS concentration increased, so did the molecular brightness, indicating the formation of larger NS/AO complex structures, which would be consistent with decreased diffusivities. Note that the FCS autocorrelation function gives us the number of diffusing species and assigns each one a molecular brightness, meaning that complexing of diffusing species will lead to a lower number of species with a higher molecular brightness. 

### 2.2. Mechanical and Physical Properties of NS-Hydrogel Composites

**Figure 4:**
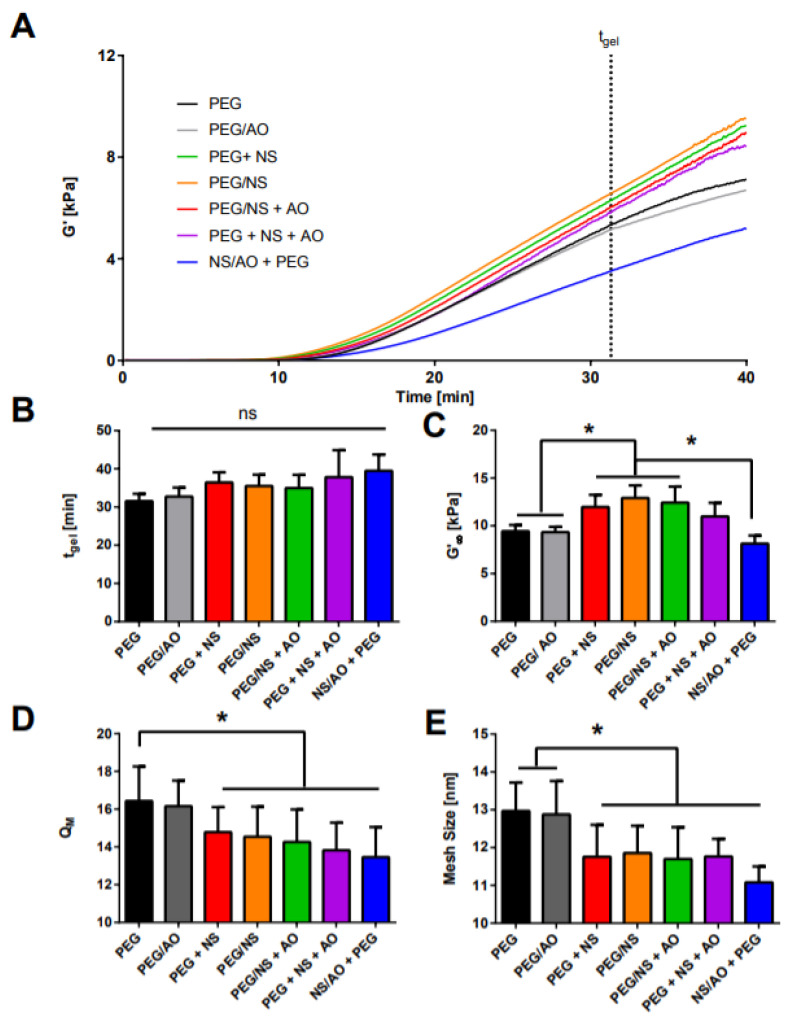
Hydrogel characterization via rheology and swelling measurements. (**A**) Evolution of *G′* over time for various combinations of PEG, NS, and AO. These data were fitted to Equation (7) to obtain *t_gel_* (**B**), which represents the time it takes to reach half of the steady-state storage modulus (*G^’^_∞_*, **C**). Swelling ratio *Q_M_* (**D**) and effective mesh size (**E**) for various combinations of PEG, NS, and AO. * designates significant differences (*n* = 3, *p* < 0.05) as measured by an ANOVA followed by Tukey’s post hoc test.

Rheology and swelling experiments were performed to characterize the interactions between NS or NS/AO complexes and the PEG hydrogel (Figure 4). Various combinations of PEG, NS, and AO were tested for their gelation time and stiffness, as measured by the storage modulus, *G′* or for their swelling and mesh size. The combinations represent mixing components together and forming a gel or pre-incubating specific components to form complexes prior to incorporation in the hydrogel. These helped us understand whether the incorporation of NS, AO, or NS/AO complexes affected hydrogel formation and structure, as well as identify possible interactions between PEG, NS, AO, and NS/AO complexes. Hereafter, the notation of “/”, such as “NS/AO”, indicates incubation for 30 min of the two species prior to addition of other components, while “+”, such as “PEG + NS”, indicates no incubation. Specifically, seven hydrogel conditions were tested: (1) PEG, which contained no NS or AO; (2) PEG/AO, where 4-arm PEG-Ac and AO were incubated for 30 min prior to adding the PEG-diSH crosslinker to initiate gelation; (3) PEG/NS, where 4-arm PEG-Ac and NS were incubated for 30 min prior to adding the PEG-diSH crosslinker to initiate gelation; (4) PEG + NS, where 4-arm PEG-Ac, PEG-diSH, and NS were mixed together with no pre-incubation; (5) PEG/NS + AO, where 4-arm PEG-Ac and NS were pre-incubated for 30 min prior to adding AO and PEG-diSH; (6) NS/AO + PEG, where NS and AO were pre-incubated for 30 min prior to adding 4-arm PEG-Ac and PEG-diSH; and (7) PEG + NS + AO, where 4-arm PEG-Ac, PEG-diSH, NS, and AO were mixed together with no pre-incubation. 

The gelation time for this timed gelation system was followed by the evolution in *G′* (Figure 4A). After ~10 min, an exponential growth of *G′* was observed for all samples, indicating gelation. This exponential growth of *G′* continued until plateauing at ~30 min for all hydrogels, indicating that a steady-state *G′* had been achieved. Using a modified Hill’s equation (Equation (7)), the gelation curves were fitted to obtain the steady-state storage modulus, *G′_∞_*, and *t_gel_*, which represents the time at which half of *G′_∞_* was achieved (Figure 4B,C). No significant differences in *t_gel_* were observed among any of the hydrogels, but differences in *G′_∞_* were noted. As expected, pre-incubating AO with PEG (PEG/AO) did not change the hydrogel stiffness, indicating that AO alone did not interfere with hydrogel formation and resulting properties. However, all hydrogels containing NS, except for NS/AO + PEG where NS was first complexed with AO, were significantly stiffer than PEG hydrogels. The NS/AO + PEG hydrogels were significantly softer than PEG. To determine whether the higher modulus was due to interactions between the NS (when not complexed with AO) and the PEG polymer, solutions of 4-arm PEG-Ac and NS or AO (no PEG-diSH to prevent gelation) were also tested via rheology (Supplemental Figure S3). These solutions showed little change in *G′* over a period of one hour, indicating no significant interactions or possible crosslinking between the NS or AO and the 4-arm PEG-Ac.

The rheology data were generally corroborated by swelling experiments. A significant decrease in the swelling ratio (*Q_M_*, Figure 4D) and mesh size (Figure 4E) were observed in all hydrogels where NS was present. This result was expected as, generally, a higher hydrogel modulus is associated with lower swelling and mesh size [35]. These results indicate that NS replaced free water molecules within the hydrogel structure, leading to lower swelling and reinforcement of the gel structure. However, the incorporation of NS/AO complexes also led to a decrease in the swelling ratio and mesh size compared to PEG-only gels, even though this group showed the lowest modulus. This could be due to the larger size of the NS/AO complexes compared to NS particles, which could be preventing efficient hydrogel crosslinking, a phenomenon typically observed when incorporating large nanoparticles into nanoporous hydrogels [36,37]. The presence of NS was shown to not have an effect on the time taken to reach equilibrium swelling (~120 min, Supplemental Figure S4). Finally, the shear-thinning behavior of NS particles PEG/NS nanocomposites were preserved upon the incorporation of AO (Supplementary Figure S5). 

**Figure 5:**
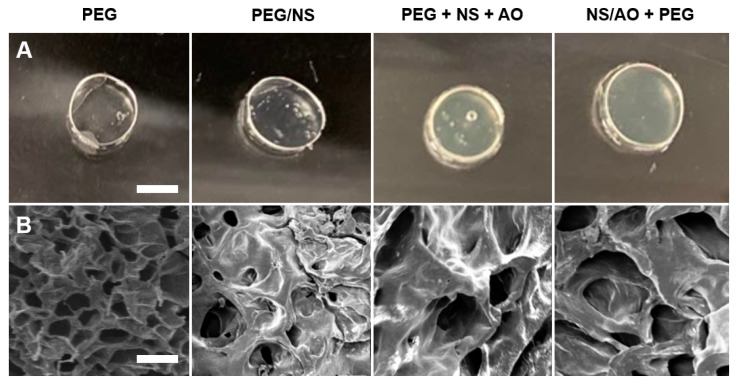
Hydrogel Morphology Characterization. Optical (**A**) and SEM (**B**) images of PEG, PEG/NS, PEG + NS + AO, and NS/AO + PEG hydrogels. Scale bars represents 10 mm (**A**) and 50 μm (**B**).

Imaging of fabricated hydrogels further revealed the effects of the presence of NS and/or AO on the hydrogel opacity and porosity (Figure 5A,B). As expected, PEG and hydrogels appeared completely transparent. PEG/NS hydrogels were also transparent as they contained fully dispersed NS particles [38]. PEG + NS + AO and NS/AO + PEG hydrogels were more opaque due to the formation of large NS/AO complexes [37]. SEM micrographs revealed the relative pore size and porosity between the hydrogels (Figure 5B, Supplemental Figure S6). PEG hydrogels showed a much higher porosity compared to NS-containing hydrogels. PEG/NS hydrogels exhibited a slightly smaller pore size than PEG hydrogels but a significantly decreased degree of porosity. This could again be attributed to NS particles replacing free water molecules within the hydrogel structure, decreasing the free volume. This further corroborates the increase in *G′* observed in Figure 4. NS/AO + PEG hydrogels showed much larger pores and decreased porosity compared to PEG hydrogels due to the formation of large NS/AO complexes disrupting crosslinking density. PEG + NS + AO represented an intermediate pore size and porosity between PEG/NS and NS/AO + PEG. 

### 2.3. Characterization of Small Molecule Diffusivity and Release from Nanocomposite Hydrogels 

**Figure 6:**
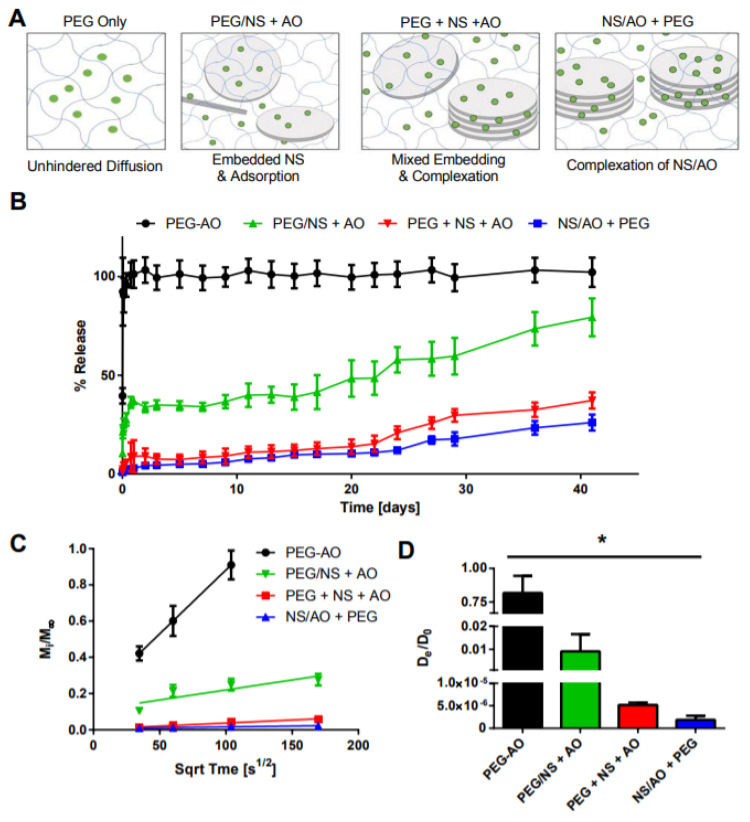
Effect of AO loading method on AO release. (**A**) Schematic of preparation conditions tested, where green dots indicate AO, blue lines represent the PEG hydrogel mesh network, and gray discs represent the NS particles. (**B**) Bulk release of AO. (**C**) Fractional release of AO as a function of the square root of time. (**D**) Calculated effective diffusion coefficient for each condition. * indicates statistically significant difference between all groups (*n* = 3, *p* < 0.05) as indicated by an ANOVA followed by Tukey’s post hoc test.

Various AO loading procedures were performed to characterize the interaction between NS and AO and its impact on the release of AO from the PEG hydrogel (Figure 6). Four preparation methods were utilized: (1) AO was added to the hydrogel precursor solution prior to gelation (PEG/AO); (2) NS was pre-incubated with 4-arm PEG-Ac for 30 min prior to addition of PEG-diSH, then the hydrogel was dried and re-swollen in a solution of AO for 24 h (PEG/NS + AO); (3) NS, AO, 4-arm PEG-Ac, and PEG-diSH were all mixed together immediately prior to gelation (PEG + NS + AO); and (4) NS and AO were pre-incubated for 30 min prior to addition of 4-arm PEG-Ac and PEG-diSH for subsequent gelation (NS/AO + PEG). These conditions represented varying degrees of availability of NS to form complexes with AO. 

In the PEG/AO group, no NS was present, representing traditional hydrogels in which the small molecule is simply encapsulated within the hydrogel and undergoes diffusion-controlled release. As expected, a significant burst release was observed for this gel, with all AO being released in the first 24 h (Figure 6B). In the PEG/NS + AO group, dispersed NS particles became embedded within the PEG hydrogel mesh, rendering them unable to form large NS/AO complexes but allowing AO to adsorb on the NS surface. For this group, ~35% of AO was released after 24 h, with release increasing to ~79% after 41 days. In the NS/AO + PEG group, NS and AO freely interacted with each other, forming NS/AO complexes that included both intercalated and surface-adsorbed AO. The PEG + NS + AO represented an intermediary between the PEG/NS + AO and NS/AO + PEG groups, where there was a competition between NS embedding and NS/AO complexation during the gelation process. This likely resulted in a mixture between individual NS particles and tactoid-like NS/AO complexes. Both NS/AO + PEG and PEG + NS + AO groups showed a substantial decrease in burst release (9% and 3% release after one day, respectively), as well as significantly prolonged release, with less than 40% of loaded AO released after 41 days. These trends were reflected in the diffusion coefficients, where AO showed the fastest diffusivity (as calculated from fractional release) in the PEG-only gels, an intermediate diffusivity when adsorbed onto the surface of NS (PEG/NS + AO condition), and the slowest diffusivity when complexed in the NS/AO + PEG condition (Figure 6C,D). 

These results indicate that, in the absence of NS, AO can rapidly diffuse out of the hydrogel matrix because of its small size. However, the incorporation of NS particles can greatly affect AO release due to electrostatic interactions and complexation between NS and AO. The timing and spatial freedom of these NS/AO interactions are critical to determining the overall release profile. When NS particles were embedded and trapped within the PEG mesh, it electrostatically adsorbed onto the charged NS surfaces. When NS and AO were allowed to form complexes prior to or during gel incorporation, AO intercalated between the NS tactoid layers, forming NS/AO complexes that greatly slowed release. 

**Figure 7:**
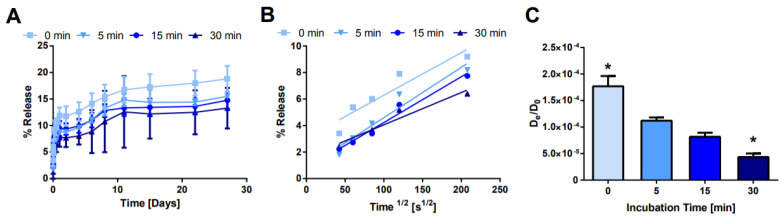
Effect of NS/AO incubation time on release. (**A**) Bulk release of AO from hydrogels with varying NS/AO incubation times with NS concentration of 1 mg/mL. (**B**) Fractional release as a function of the square root of time. (**C**) Effective diffusion coefficient for each condition. * indicates statistically significant difference from all other groups (*n* = 3, *p* < 0.05) as indicated by an ANOVA followed by Tukey’s post hoc test.

Next, the NS/AO incubation time was modulated to observe the kinetics of NS/AO complexation (Figure 7). NS and AO were mixed for 0–30 min prior to the addition of four-arm PEG-Ac and PEG-diSH (NS/AO + PEG condition). As expected, the longer the incubation time between NS and AO, the slower the release from the PEG hydrogels. After 28 days, ~19% of the encapsulated AO was released from the 0 min incubation hydrogels compared to ~12% for the 30 min incubation condition. Furthermore, the burst release was reduced as the incubation time increased. Similar trends were observed when the NS concentration was increased from 1 to 10 mg/mL (Supplemental Figure S7). It should be noted that for all incubation times studied here, the burst release was minimized and the overall release was substantially prolonged compared to PEG (no NS) hydrogel controls. 

**Figure 8:**
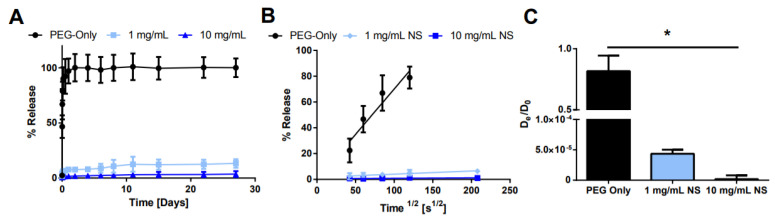
Effect of NS concentration on release. (**A**) Bulk release of AO from hydrogels with varying NS concentration. (**B**) Plot of fractional release as a function of the square root of time. (**C**) Calculated effective diffusion coefficient for each condition. * denotes statistically significant difference between all groups (*n* = 3, *p* < 0.05) as indicated by an ANOVA followed by Tukey’s post hoc test.

Next, the NS concentration was modified while keeping the AO concentration constant to determine if the additional NS surface area led to longer sustained release (Figure 8). As expected, as the NS concentration increased, the burst release was reduced and the overall AO release was prolonged. For 1 mg/mL NS, ~13% of the loaded AO was released after 28 days, while only ~3.7% of AO was released from the 10 mg/mL NS hydrogels. 

**Figure 9:**
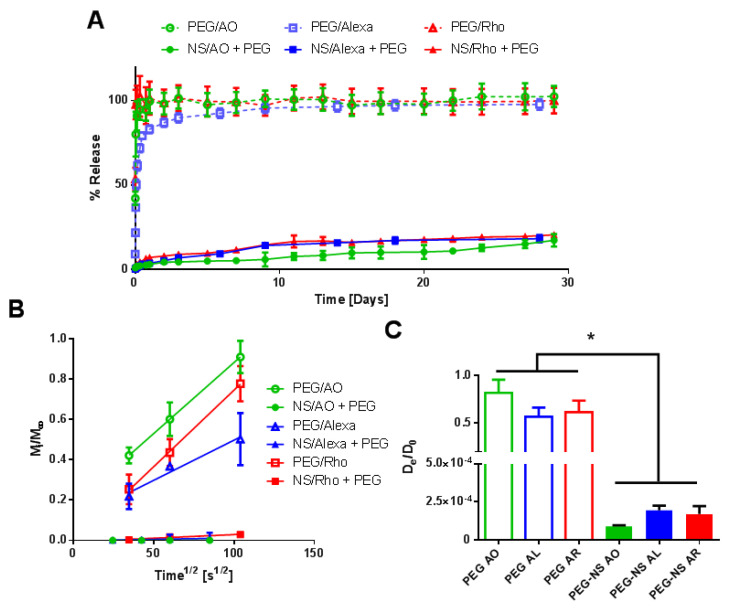
Release of varying model small molecules. (**A**) Bulk release of AO, Alexa, and Rho from hydrogels with 30 min incubation time and NS concentration of 0 mg/mL (PEG) or 1 mg/mL (PEG-NS). (**B**) Plot of fractional release as a function of the square root of time. (**C**) Calculated effective diffusion coefficient for each condition. * denotes statistically significant difference between all groups (*n* = 4, *p* < 0.05) as indicated by an ANOVA followed by Tukey’s post hoc test.

Here, we showed how the preparation conditions, pre-incubation with NS, as well as the NS concentration could be used to tune release of AO from PEG/NS hydrogel composites. To demonstrate that the results obtained were not specific to AO, two other model small molecules were used in key release studies: Alexa 647 and Atto Rho 13, abbreviated here as Alexa and Rho, respectively. Both molecules are fluorophores with a net positive charge at neutral pH, similarly to AO (Table 1). Here, a 30 min NS–small-molecule incubation time and NS concentration of 1 mg/mL as these conditions were previously shown to lead to minimal burst release and prolonged release (Figure 9). 

Similar release profiles between all three small molecules were observed both in the absence and presence of NS particles. Without NS to interact with the small molecules, >85% of the loaded small molecule was released within the first 24 h for all three model small molecules. However, the inclusion of NS significantly minimized burst release (<7% release after one day) for all three small molecules. The slow release continued, with <25% release of all three small molecules at 30 days. Additionally, similar trends were observed when the various preparation methods mentioned in Figure 3 were used with Alexa and Rho (Supplemental Figures S8 and S9). Thus, the interaction between small molecules and NS was not specific to AO but was present in other model small molecules, as well as therapeutics, as seen by others [19,20]. 

## 3. Discussion

Hydrogels are widely used for therapeutic delivery due to their biocompatibility, biodegradability, and ability to control therapeutic release [39]. Specifically, PEG hydrogels have been shown to be bio-inert and non-cytotoxic [40,41]. However, most hydrogels are susceptible to loss in therapeutic bioactivity, high initial burst release, and ineffectiveness of sustained delivery of small molecules [42]. The addition of nanoparticles to polymeric hydrogels has been shown to improve the delivery kinetics and alleviate burst release due to increased retention [43]. Specifically, disc-shaped two-dimensional NS have been exploited for release applications by themselves and in nanocomposite hydrogel systems [12] because they are biocompatible, biodegradable, and noncytotoxic [44]. The high surface area and charge characteristics of NS particles can be used to electrostatically adsorb a range of small molecules to form NS–small-molecule complexes, offering the strong potential for a robust, ‘plug-and-play’ type of delivery device [21,45]. However, little is known about the structure and size of NS–small-molecule complexes on their effect on composite hydrogel properties and release kinetics. 

The burst release of small molecules from traditional hydrogels limits the utility of these hydrogels as sustained delivery devices. Small molecules, which have sizes significantly smaller than the effective mesh sizes of hydrogels, can easily diffuse out of the hydrogels if no hydrogel–small-molecule interactions are present. The incorporation of a nanomaterial such as NS can serve to alleviate such rapid burst release by allowing for small molecule adsorption, intercalation, or tethering [12]. Thus, incorporating NS in hydrogels offers another lever by which release can be controlled, offering enhanced tunability of release profiles [46]. However, knowledge of the mechanisms of small-molecule–NS interactions is crucial for fine-tuning and controlling small-molecule release profiles. 

Here, AO, Alexa, and Rho were used as model small molecules because of their size, positive net charge at physiologic pH, and intrinsic fluorescence, allowing for ease of detection (Table 1). These model molecules allowed us to elucidate the kinetics of NS/small molecule interactions and the resultant structures. Cationic small molecules were used here due to their ability to interact with the negatively charged faces of NS particles, offering more surface area than the positively charged NS particle edges [15]. AO was used for all experiments and Alexa and Rho were used for key release experiments to confirm that our findings were not confined to AO only but could be generalized to other similar small molecules. NS was used because of its demonstrated ability to slow release of a wide range of molecules, including small molecules [20], therapeutics [47], and proteins [37]. NS has unique surface-area and surface-charge characteristics, with a high surface-area-to-volume ratio due to its two-dimensionality as well as positively charged edges and negatively charged faces. PEG was used as the hydrogel backbone because of its tunability, biocompatibility, and bio-inertness [48]. 

We first sought to elucidate the mechanism of NS/AO interaction. XRD revealed that AO most likely intercalates between layers of NS in the (001) plane, as evidenced by an increase in *d*-spacing (Figure 1A). Native sodium ions experience cationic exchange with AO molecules, where AO replaces the intercalated sodium ions on the negatively charged faces of NS particles [22]. Such an increase in *d*-spacing when NS was mixed with AO was expected due to the larger size of AO compared to a single sodium ion, (~0.5 nm and 227 pm, respectively) [49]. The size of the *d*-spacing in the NS/AO sample may be indicative of a bilayer of AO in the interlayer space, though further investigation must be performed to determine the influence of NS and AO concentration on the specific interaction orientations [22]. Similar changes in the (001) plane have been observed with other small molecules, including doxorubicin and tetracycline [19,20,47,50]. The lack of change of spacing in the other NS planes indicated that the NS crystalline structure was otherwise maintained [47]. 

FTIR-ATR further confirmed the interaction between NS/AO (Figure 1B). Peaks indicative of AO were clearly present in the NS/AO spectrum, notably at 1150 cm^−1^ and 1401 cm^−1^. These results seemingly indicated interaction of AO with the Si-O-atoms of NS, as evidenced by the shift in the peak near 991 cm^−1^. Such a result was to be expected, as Si-O-atoms reside near the faces of the NS particles, further corroborating the hypothesis that AO was interacting with the NS particle faces, leading to the intercalation and formation of tactoid-like structures. The intercalation of AO between NS layers also partially shielded AO from degradation, as seen via TGA (Figure 2). While free AO exhibited ~50% mass loss below 500 °C, less than 10% mass loss was observed for NS/AO. The multiple mass loss peaks present in the NS/AO plot presumably represent AO with varying degrees of shielding from degradation by NS. Therefore, we suggest that, in addition to the intercalation of AO between NS tactoid layers, some AO was also adsorbed to the surface of NS, making it more susceptible to thermal degradation. The intercalated AO resisted mass until decomposition of the crystal structure of NS, which further corroborates the hypothesis of intercalation. 

With the knowledge of the mechanism of NS/AO interactions, the next step was to determine the effects of NS, AO, or NS/AO complexes on hydrogel gelation, mechanical properties, and swelling behavior. Degradation of hydrogel delivery devices via hydrolytic or enzymatic degradation can be crucial in determining release profiles, as release can be affected by the integrity and swelling of the hydrogels [2]. Stiffer hydrogels may be indicative of a higher degree of crosslinking, which can obstruct small-molecule release by decreasing effective mesh size, thereby creating a more tortuous path through which diffusion must proceed [51]. The incorporation of nanoparticles within the hydrogels may serve to increase the stiffness due to enhanced solid content, or may disrupt crosslinks, leading to a softer hydrogel [52].

The effects of NS particles and NS/AO complexes on hydrogel gelation were studied via rheology (Figure 4). NS particles seemingly reinforced the hydrogel mesh structure, leading to an increase in steady-state *G′* [53]. As no significant increase in *G′* was observed when mixing four-arm PEG-Ac and NS without PEG-diSH (Supplemental Figure S3), it is unlikely that the NS particles were acting as secondary crosslinkers. Rather, we suggest that the NS particles acted as crowders that decreased the free volume within the hydrogel mesh, increasing the solid content within the hydrogel [29,54,55]. This is corroborated by the decreased swelling ratio and mesh size in all hydrogels containing NS, as well as decreased pore size and porosity (Figure 5). Similar swelling ratios and mesh sizes for PEG hydrogels have been shown by others [35,56,57]. On the other hand, the formation of NS/AO complexes led to a decrease in steady-state *G′*. This could be attributed to the sizes of NS/AO complex structures, which may be reminiscent of the tactoid structures of NS powder, preventing the formation of a congruent hydrogel mesh network by decreasing the crosslinking density, as observed via SEM (Figure 5). Thus, a significant difference in mechanical properties was present between NS particles only and NS/AO complexes. For the PEG + NS + AO group, for which NS and AO were not incubated together prior to addition of PEG, a competition proceeded between the formation of NS–AO complexes and gelation, in which NS particles become embedded within the mesh network and are therefore unable to form complexes with AO and other NS particles. Note that the differences between mesh size measured via swelling and the pore size measured via SEM can be attributed to the freeze-dried state of the hydrogels for SEM imaging [58].

The release of AO from these nanocomposite hydrogels was affected by both NS/AO complexation and the effect of NS, AO, or NS/AO complexes on hydrogel properties. AO release could be attributed to the desorption of AO from NS surfaces, de-intercalation of AO from NS interlayer spaces, or degradation of NS particles. Different NS/AO preparation methods were utilized to observe the impacts on AO release profiles (Figure 6). The PEG-only group, which contained no NS, saw a significant burst release, as AO release was diffusion-controlled. Since the size of AO molecules was significantly smaller than the calculated mesh size (~0.5 nm and ~11–13 nm, respectively), AO could diffuse mostly freely through and out of the hydrogels [59]. Rapid release of small molecules within hours has been seen by others from PEG-only hydrogels [60]. This fast release of AO served as the motivation for incorporating NS into the hydrogels due to their demonstrated ability to intercalate within and adsorb to the surface of NS particles. Note that while not studied here, the PEG hydrogels used here were expected to undergo hydrolytic degradation of the thioester bond, resulting in a decrease in crosslink density and stiffness, leading to an increase in swelling ratio and effective mesh size over time [3,61].

In the PEG/NS + AO group, individual NS particles were first embedded within the hydrogel mesh network. Note that the size of NS particles (~35 nm) was approximately three times larger than the effective gel mesh size (~11–13 nm), so they became entrapped in the hydrogel mesh upon gelation. This rendered the NS unable to form larger NS/AO complexes upon AO addition, but AO was still able to absorb onto the NS surfaces (Figure 6A) [18,62]. Hence, AO release was significantly higher than the other NS-containing groups where NS/AO complexes could be formed, but burst release was alleviated and release was prolonged compared to the PEG-only group. Furthermore, a higher AO release rate was observed after ~20 days compared to the PEG + NS + AO and NS/AO + PEG groups. This could be indicative of the degradation of individual NS particles which are known to degrade in 30–50 days [16], possibly leading to desorption and release of AO.

For the NS/AO + PEG group, NS and AO were incubated together for 30 min prior to addition of PEG, therefore allowing for the two species to interact and form complexes with adsorbed and intercalated AO. These NS/AO complexes were then encapsulated within the hydrogels, effectively holding AO within the hydrogels. As discussed above, the PEG + NS + AO group served as an intermediary between the PEG/NS + AO and NS/AO + PEG groups. The kinetics of the formation of complexes of NS and AO was in competition with that of gelation, in which induvial NS particles would become embedded within the hydrogel mesh network. As NS was not as free to form complexes with AO in the PEG + NS + AO group, slightly higher burst release was observed compared to the NS/AO + PEG group. 

The burst release in the NS-containing groups could be attributed to AO molecules that did not interact with NS particles, allowing for rapid diffusion and release out of the hydrogel. For example, we saw that even with an excess of NS and 30 min incubation, only 92% of AO was adsorbed (Figure 3D). Using the literature value of 0.140 mmol/g as the cation exchange capacity of NS [31] and the concentration of AO used for these FCS studies (0.02 μM), we calculated an excess of NS charge “binding” sites above NS concentration of 0.001 mg/mL. Below this NS concentration, an excess of AO was present, leading to incomplete adsorption/intercalation and reduced bound percentage, as seen here. Remaining AO likely interacted with NS particle either through adsorption or intercalation, effectively holding the AO within the hydrogel [19]. As time progressed, desorption or de-intercalation of AO also progressed, freeing AO molecules to subsequently be rapidly released. The desorption and de-intercalation could be caused by the same process that led to NS/AO interactions: cationic exchange. The cationic ions present in the release sink solution of PBS could reversibly exchange places with the adsorbed/intercalated AO, releasing AO [63]. 

The relationship between NS/AO incubation time was further explored (Figure 7, Supplemental Figure S7). As the incubation time increased, the burst release was reduced and the overall release was prolonged. These data suggest that the interaction between NS and AO and formation of NS/AO complexes is not instantaneous. A similar kinetic assembly of NS-containing complexes has been observed previously by us and others when NS was mixed with proteins [37,64]. This could be due to NS particles re-forming their tactoid-like structure with intercalated AO due to cation exchange, or the formation of a more complicated and ambiguous complex structure. A maximum incubation time of 30 min was used here to prevent physical gel formation by NS particle–particle interactions at the relatively high NS concentrations used prior to dilution by PEG and TEA [65]. 

The concentration of NS was also shown to have an effect on AO release from hydrogels as measured by bulk release studies (Figure 7) and diffusivity in solution as measured by FCS (Figure 3). Higher NS concentrations led to slower AO release and reduced AO diffusivities compared to lower NS concentration. This is because the higher number of NS particles presented additional surface area to which AO could adsorb as well as more intercalation of AO in the NS tactoid. The diffusivity data were corroborated with complex size and brightness data, wherein an increase in NS concentration led to the formation of larger (hence, slower diffusing) and brighter (due to more fluorescent AO) NS/AO complexes. It should be noted that the NS concentrations used for FCS were much smaller than those utilized in the release studies. This was due to large aggregates forming at higher NS concentrations, which led to large intensity spikes that prevented accurate and repeatable results. These aggregates may have represented large NS tactoid structures that remained non-exfoliated during NS dispersion, or abnormally large NS/AO complexes. Additionally, a relatively short measurement time was utilized (120 s) for AO measurements to prevent photobleaching of AO. 

Lastly, release tests were performed with Alexa and Rho to confirm the repeatability of results with other small molecules, and each exhibited similar release profiles for all conditions tested (Figure 9 and Supplemental Figures S8 and S9). The release kinetics were also relatively similar to other small molecules from individual NS particles by themselves [19,47] or from other NS-composite materials [26,27], which show that release in the presence of NS significantly slowed. The various preparation conditions explored here can serve as the basis for creating a “library” of release profiles that could be utilized to fine-tune release of small molecules for targeted applications. By modulating NS/small molecule preparation conditions, NS/small molecule incubation time, and NS concentration, we could tune the small molecule release profile from a few hours to a period of over a month. Such versatility is critical for development of a robust delivery device that can be used for a wide variety of applications. 

## 4. Materials and Methods

### 4.1. Materials

Four-arm PEG-Acrylate (4-arm PEG-Ac; 10 kDa) and PEG-dithiol (PEG-diSH; 3.4 kDa) were purchased from Laysan Bio Inc. (Arab, AL, USA). Acridine Orange, Alexa 647, Atto Rho 13, and triethanolamine (TEA) were obtained from Millipore Sigma (Saint Louis, MO, USA). Nanosilicate particles (Laponite XLG, abbreviated here as NS) were obtained from BYK Additives (Wesel, Germany). CoverWell perfusion chamber gaskets and silicone spacers were procured from Grace Bio-Labs (Bend, OR, USA). 

### 4.2. Characterization of NS–AO Interactions

The powder diffraction patterns of NS particles and NS–AO complexes were measured using Rigaku XRD MiniFlex 600 (Tokyo, Japan) from 2θ of 2–90° with CuKα radiation and step size of 0.02°, voltage of 40 kV, and current of 30 mA. NS–AO samples were prepared by mixing 10 mg/mL NS and 0.1 mM AO for 30 min. Samples were then centrifuged (1500 rpm for 5 min) and freeze dried prior to use. The *d*-spacing of the basal (001) layers of NS particles with and without AO was calculated using Bragg’s equation [22]:

(1) λ=2dsinθ

where *λ* is the wavelength of CuKα radiation (1.540 Å), *d* is the interlayer spacing of NS, and *θ* is the scattering angle. 

Fourier transform infrared (FT-IR) spectroscopy with attenuated total reflectance (ATR) was also utilized (Shimadzu FTIR-8400S with ATR accessory, Tokyo, Japan). NS-only and NS–AO complexes were prepared as described above (10 mg/mL NS and 0.1 mM AO). 

Thermogravimetric analysis (TGA) was performed using a TA Instruments SDT Q600 DSC/TGA (New Castle, DE, USA). The temperature was increased from 25 °C to 900 °C at a rate of 10 °C/min with inert argon flowing at a rate of 50 mL/min.

### 4.3. Fluorescence Correlation Spectroscopy

Fluorescence correlation spectroscopy (FCS) was performed using a Microtime200 confocal microscope and associated PicoQuant Software (Berlin, Germany) and was used to measure AO diffusivity in solution as a function of NS concentration. Acridine orange powder was dispersed in DI water at a concentration of 1 μM, while NS was prepared in DI water at concentrations ranging from 0.0001 mg/mL to 0.1 mg/mL. For each measurement, 1 μL of 1 μM AO was added to a tube containing 99 μL of NS of specified concentration and allowed to incubate for 30 min. AO–NS solutions (40 μL) were pipetted into a perfusion chamber gasket adhered to a #1.5 coverslip and capped to avoid evaporation. FCS measurements utilized a 532 nm ps pulsed laser at an optical power of ~11 μW for at least five separate measurements of 120 s for each sample. 

An autocorrelation function, *G(τ)*, was obtained for each measurement using the PicoQuant software [66]:

(2) $$G\left(\tau \right)=\frac{1}{N}\frac{1}{\left[1+\left(\frac{\tau }{\tau_{D}}\right)\right]}\frac{1}{{\left[1+p\left(\frac{\tau }{\tau_{D}}\right)\right]}^{0.5}}$$

where *N* is the number of fluorescent particles, *p* = *r_o_*/*z_o_* is an instrumental constant, *r_o_* is the radius and *z_o_* is the axial length of the focused laser beam spot, and *τ_d_* is the solute diffusion time. For two non-interacting, diffusing solutes, Equation (2) can be re-written as [67,68]:

(3) $$G\left(\tau \right)=1+m_{1}\frac{1}{\left[1+\left(\frac{\tau }{\tau_{1}}\right)\right]}\frac{1}{{\left[1+p\left(\frac{\tau }{\tau_{1}}\right)\right]}^{0.5}}+m_{2}\frac{1}{\left[1+\left(\frac{\tau }{\tau_{2}}\right)\right]}\frac{1}{{\left[1+p\left(\frac{\tau }{\tau_{2}}\right)\right]}^{0.5}}$$

where *m*_1_ and *m*_2_ are related to the quantum yield and average number of each diffusing species and *τ*_1_ and *τ*_2_ are their respective diffusion times. Each autocorrelation function was fitted using a triplet model to account for the excitation of molecular triplet states at higher laser intensities. Furthermore, autocorrelation functions were normalized using the equation:

(4) $$Normalized\;G\left(\tau \right)=\frac{G\left(\tau_{D}\right)}{G\left(\tau_{0}\right)}$$

where *G(τ_D_)* is the value of the Equation (3) at each time point and *G(τ*_0_) is the value of Equation (3) at the initial time point. The effective diffusion coefficient (*D_FCS_*) for AO in solution was calculated from *τ_D_* as [68]:

(5) $$D_{FCS}=\frac{{\left(r_{0}\right)}^{2}}{4\tau_{D}}$$

AO in DI water was used as a control for these experiments and was used to calibrate the confocal volume (1.043–1.232 fL) because of its known diffusion coefficient [69]. Therefore, *τ_D_* for free, unbound AO was measured using a single component fit (Equation (2)) and used as a fitted parameter (*τ*_2_ in Equation (3)) to determine the diffusion time of AO adsorbed onto NS.

The radius of the slow-diffusing species was calculated using the Stokes-Einstein equation:

(6) $$r_{s}=\frac{k_{B}T}{6\pi \eta D_{FCS}}$$

where *k_B_* is the Boltzmann constant (1.3806 × 10^−23^ m^2^ kg/s^2^ K), *T* is the temperature (310 K), *η* is the viscosity of water (8.9 × 10^−4^ Pa s), and *D_FCS_* was the diffusion coefficient calculated using Equation (5). 

### 4.4. Hydrogel Fabrication 

Hydrogels were formed using Michael-type addition using 4-arm PEG-Ac and PEG-diSH. Stock solutions of 200 mg/mL 4-arm PEG-Ac and 200 mg/mL PEG-diSH were prepared in 0.3 M triethanolamine (TEA, pH 7.4). NS particles were dispersed in deionized water at a concentration of 30 mg/mL, as previously described [37]. This NS solution was then added to the 4-arm PEG-Ac precursor solution for a final NS concentration of 10 mg/mL. PEG-diSH was then added to the 4-arm PEG-Ac/NS solution to obtain an equimolar ratio of acrylate: thiol end groups and a final PEG concentration of 10 mg/mL. The hydrogel solution was then pipetted between two glass slides covered with Parafilm, with 1 mm silicone spacers at each corner of the glass slides. Hydrogels were allowed to undergo gelation for ~60 min. 

### 4.5. Rheological Measurements 

All rheological measurements were performed on an AR2000ex Rheometer (TA Instruments, New Castle, DE, USA). To evaluate the hydrogel gelation time, the storage modulus, *G′*, was followed over time at a constant strain of 2% and angular frequency of 10 rad/s at room temperature using a parallel-plate geometry. The evolution in *G′* was fit to a modified Hill Equation to calculate the effective gelation time [70]:

(7) $$G^{\prime }\left(t\right)={G^{\prime }}_{\infty }\left(\frac{1}{1+{\left(\frac{t_{gel}}{t}\right)}^{m}}\right)$$

where *t_gel_* is the time required to reach half of the steady-state storage modulus (*G′_∞_*) and *m* is the Hill coefficient related to the slope of the gelation curve. The final PEG concentration was 100 mg/mL, the final NS concentration was 10 mg/mL, and the final AO concentration was 0.03 mM. 

To measure the effect of shear rate on hydrogel precursor solution viscosity, hydrogel precursor solutions were pipetted onto the rheometer stage and allowed to equilibrate for 60 s. Viscosity was measured at shear rates of 0.01–100 s^−1^ and a constant strain of 2%. The Ostwald–de Waele relationship was used to calculate values of the flow consistency index, *K*, and the flow behavior index, *n*, using the following equation, where *η* is the measured viscosity and *γ* is the shear rate [71]:

(8) $$\eta =K\gamma^{n-1}$$

### 4.6. Hydrogel Swelling and Mesh Size

PEG hydrogels with and without NS and AO were formed as described above in a slab geometry with a final volume of 30 µL per gel. The initial mass, *M*_0_, of each gel was measured using a Mettler Toledo XS104 Balance (Columbus, OH, USA). Hydrogels were then incubated in 1× Phosphate Buffered Saline (PBS) for 24 h at 37 °C. Gels were removed from the PBS and excess liquid was removed by gently patting with a Kimwipe. The swollen mass, *M_S_*, of the gels was then measured. The dry mass, *M_D_*, was obtained by weighing each gel following 24 h of drying in a 60 °C oven. The swelling ratio, *Q_M_*, was calculated as:

(9) $$Q_{M}=\frac{M_{s}}{M_{D}}$$

Furthermore, the kinetics of initial hydrogel swelling were observed by following the percentage initial mass until equilibrium swelling was obtained:

(10) $$\%\;\mathrm{Initial}\;\mathrm{Mass}=\frac{M_{s}}{M_{0}}$$

where *M*_0_ is the initial hydrogel mass (as fabricated) and prior to swelling in buffer. 

The average mesh size, *ξ*, of each gel was calculated using the Flory–Rehner theory [3,59]:

(11) $$\xi ={\left(\nu_{2,s}\right)}^{-\frac{1}{3}}{\left(\frac{2C_{n}{\bar{M}}_{c}}{M_{r}}\right)}^{\frac{1}{2}}l$$

where *ν*_2,*S*_ is the polymer volume fraction in the swollen state, *C_n_* is the characteristic ratio for the polymer (4 for 4-arm PEG-Ac), *M_C_* is the average molecular weight between crosslinks, *M_r_* is the molecular weight of a polymer repeat unit (44 g/mol for PEG), and *l* is the average bond length (1.46 nm). 

### 4.7. Scanning Electron Microscopy

To visualize the hydrogel morphology, hydrogels were soaked in DI water for 24 h, frozen at −80 °C for 1 h, and lyophilized for 24 h (Lyophilizer, VirTris Sentry 2.0, Warminster, PA, USA). Samples were then sputter coated with gold (SCD 005, Bal-TEC, Balzers, Liechtenstein) and imaged using EVO LS15 SEM (Zeiss, Thornwood, NY, USA) at 5 kV, under high vacuum, at a magnification of 200×. The pore size and porosity were measured using NIH ImageJ software. The pore size was estimated by measuring the long axis of at least 50 pores per sample [72,73]. To analyze the porosity, SEM images were first binarized, then the porosity was calculated from the areas of black (pores) and white (hydrogel wall) pixels [74]. 

### 4.8. Bulk Release Studies

To optimize the hydrogel fabrication process for sustained release, various preparation methods were explored using AO as a model small molecule. The incubation time of NS particles and AO was varied between 0 and 30 min prior to the addition of PEG precursor solutions to elucidate the kinetics of electrostatic adsorption of AO onto NS. Separately, the NS concentration was varied between 0 and 10 mg/mL NS to observe the effect of NS concentration on AO release profiles.

Using the optimized preparation conditions of 30 min of incubation time and 10 mg/mL NS, a bulk release study was performed with AO, Atto Rho13, and Alexa 647 as model small molecules. Final hydrogel concentrations were as follows: 0.1 mM small molecule, 10 mg/mL NS, and 100 mg/mL PEG. Hydrogels were formed as described above with a final volume of 30 μL and dimensions of 15 mm in diameter and 1 mm in height. 

After 60 min of gelation time, hydrogels were removed from glass slides, placed in 1.5 mL microcentrifuge tubes with 1000 μL of 1X PBS at 37 °C, and incubated with shaking on a shaker platform (Versa-Orb Oribital Shaker, Chemglass Life Science, Vineland, NJ, USA). At specified time points, 250 μL aliquots were removed from each tube and 250 μL of fresh 1X PBS was added back to each tube to return the sink volume to 1000 μL. The small-molecule concentration was determined by measuring the fluorescence of releasate samples at the wavelengths described in Table 1 using a SpectraMax i3 plate reader (Molecular Devices, San Jose, CA, USA). 

A mass balance was performed to calculate the total mass of released protein at each time point as:

(12) $$\frac{M_{i}}{M_{inf}}=C_{i}V+\sum C_{i-1}V_{s}$$

where *M_i_* is the concentration of protein released at time *i*, *M_inf_* is the concentration of protein at infinite time, *M_i_*/*M_inf_* is the fractional release, *C_i_* is the concentration of protein in the releasate at time *i*, *V* is the total volume of the release solution, and vs. is the releasate sample volume. The effective diffusion coefficient was calculated for short release times using a modified form of Fick’s law [75]:

(13) MiMinf=2[Detπδ2]12

where *D_e_* is the effective diffusion coefficient, *t* is the release time, and *δ* is half of the hydrogel thickness (0.5 mm). The effective diffusion coefficient was then normalized by the diffusivity of the small molecule in water, *D*_0_, at 37 °C, given in Table 1.

## 5. Conclusions

In conclusion, the release of AO was significantly reduced in the presence of NS within PEG/NS composite hydrogels due to the surface adsorption and intercalation of AO between the NS layers leading to the formation of NS/AO complexes. The formation of these NS/AO complexes was confirmed using XRD, TFA, FTIR, and FCS, revealing an interaction between the cationic AO and negatively charged surface of NS particles. NS/AO complexation played a crucial role in governing AO release kinetics. When individual NS particles were embedded within the PEG, AO could adsorb to the NS surface, resulting in moderately slowed release (70-fold compared to PEG-only hydrogels). When NS was first pre-incubated with AO to form large NS/AO complexes where AO adsorbed onto NS and intercalated between the NS tactoid layers, AO release was slowed >9000-fold compared to PEG-only hydrogels. The NS concentration and NS/AO incubation time were also shown to affect the bulk release profiles due the formation of larger NS/AO complexes. By modulating the parameters discussed here, a variety of release profiles could be obtained, offering tunability and the potential for a controlled-release plug-and-play device. 

## Figures and Tables

**Table 1:** Properties of model small molecules used in this study.

Small Molecule	Acridine Orange	Alexa 647	Atto Rho 13
Abbreviation	AO	Alexa	Rho
Molecular Weight [g/mol]	265	1025	867
Diffusion Coefficient in Water at 37 °C [×10^−6^ cm^2^/s]	8.7	3.3	4.6
Ex./Em. Wavelength [nm]	500/540	640/670	590/630
Net Charge at pH 7.4	+1	+1	+1
Chemical Structure	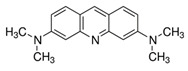	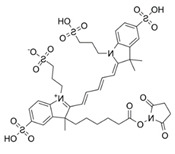	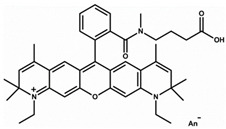

## Data Availability

The data are contained within the article or supplementary materials.

## Supplementary Materials

The following are available online at https://www.mdpi.com/article/10.3390/ph15010056/s1: Figure S1: FTIR-ATR of AO, Figure S2: Differential Scanning Calorimetry, Figure S3: Rheology of Hydrogel Precursor Solutions, Figure S4: Swelling Kinetics of PEG Hydrogels, Figure S5: Shear-thinning Behavior of NS is Retained, Figure S6: Porosity of Hydrogels Measured with SEM, Figure S7: Influence of NS/AO Incubation Time with 10 mg/mL NS, Figure S8: Release of Alexa 647, Figure S9: Release of Atto Rho 13.
