# Supplementary material for: Adsorption and Sustained Delivery of Small Molecules from Nanosilicate Hydrogel Composites
Source: Pharmaceuticals (Basel). 2022 Jan 1;15(1):56. doi: 10.3390/ph15010056 (PMC8780425; doi:10.3390/ph15010056)
Supplement: Supplementary file 1 [file pharmaceuticals-15-00056-s001.zip › pharmaceuticals-1522323-supplementary.pdf]

# Supplemental Data

# Adsorption and Sustained Delivery of Small Molecules from Nanosilicate Hydrogel Composites


Samuel Stealey [1], Mariam Khachani [1] and Silviya Petrova Zustiak [1,*]

[1]Biomedical Engineering Program, Parks College of Engineering, Saint Louis University, Saint Louis, MO, USA 63103

*Correspondence: silviya.zustiak@slu.edu; Tel.: 1-314-977-8331


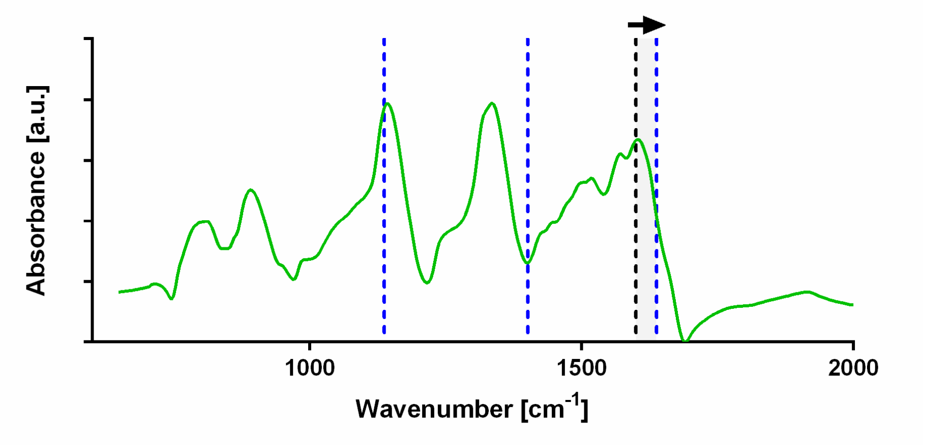


**Supplemental Figure S1: FTIR-ATR of AO**. Spectrum of Acridine Orange (AO) in the absence of NS. Drop lines represent regions indicative of NS/AO complexes at 1127, 1401, and 1633 cm$^{-1}$, as described in **Figure 1C**. Black drop line indicates band at 1603 cm$^{-1}$ that is typical of neutral AO, which is shifted to 1633 cm$^{-1}$ in cationic $AOH^{+}$.

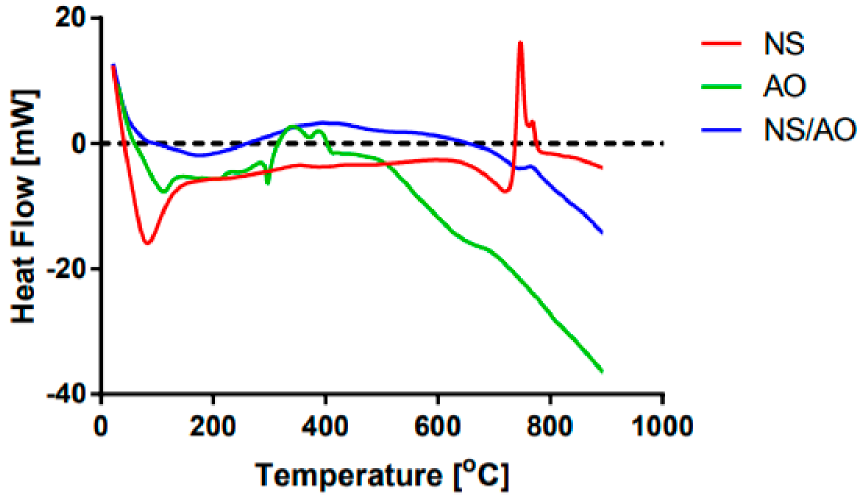


**Supplemental Figure S2: Differential Scanning Calorimetry**. DSC measurements of NS, AO, and NS/AO performed in tandem with TGA measurements (**Figure 2**).

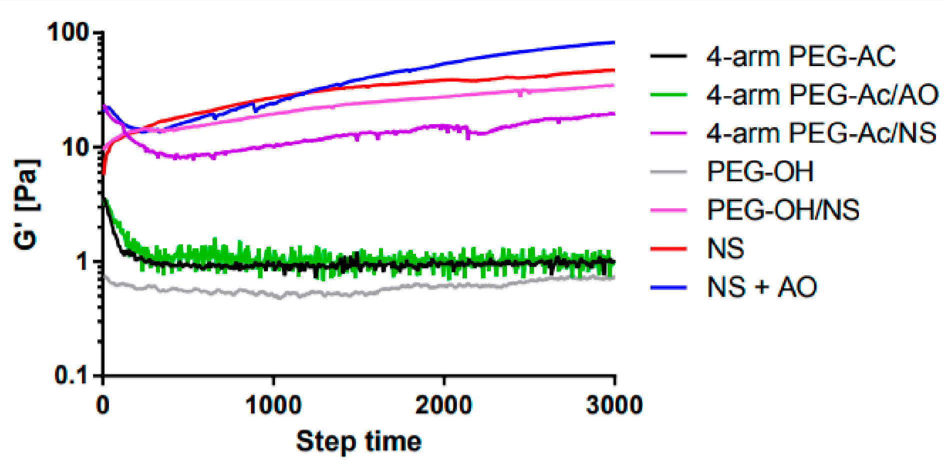


**Supplemental Figure S3**: **Rheology of Hydrogel Precursor Solutions**. Measured *G'* of hydrogel precursor solutions (no added PEG-diSH) measured over time.

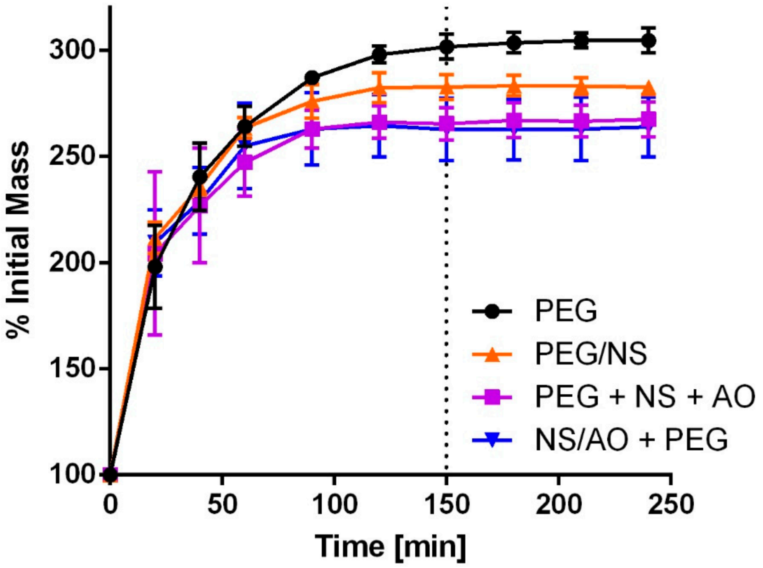


**Supplemental Figure S4**: **Swelling Kinetics of PEG Hydrogels**. Measured percent initial mass of hydrogels in the absence or presence of NS and/or AO.

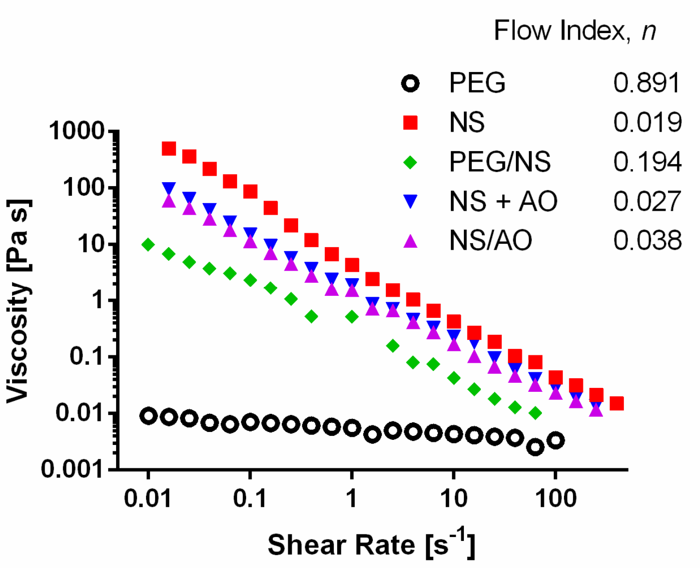


**Supplemental Figure S5: Shear-thinning Behavior of NS is Retained.** Shear rate sweeps of various combinations of PEG, NS, and AO reveals preservation of NS shear-thinning behavior. Data were fitted to **Eq. 8** obtain Flow Index, *n*. An *n* value of < 1 indicates shear-thinning behavior.

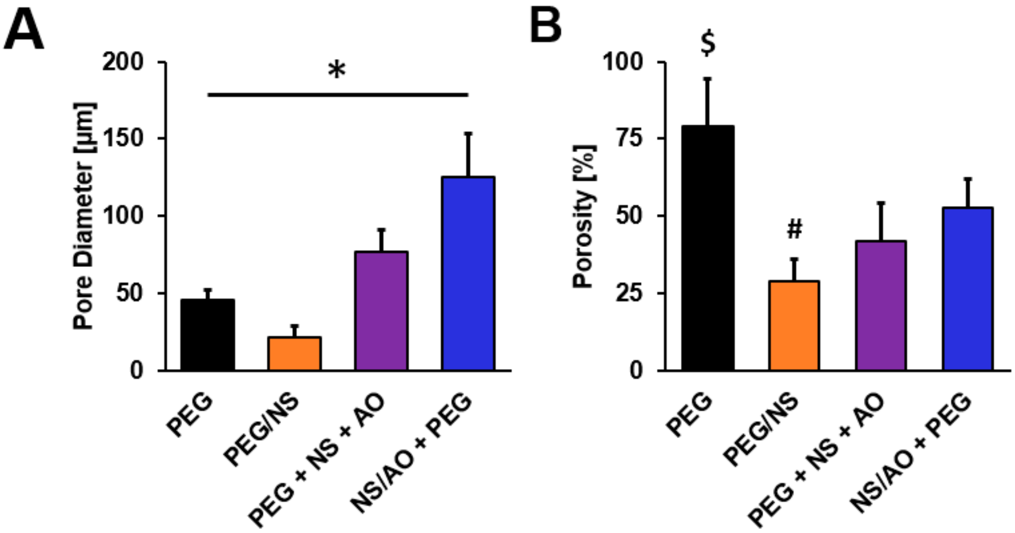


**Supplemental Figure S6: Porosity of Hydrogels Measured with SEM. A**. Pore diameter (**A**) and porosity (**B**) of hydrogels with different combinations of PEG, NS, and AO. Hydrogels were visualized with SEM following lyophilization. * indicates significant difference between all groups ($p < 0.05$, N = 3). $^{\$}$ indicates significant difference from all other groups, while $^{\#}$ indicates significant difference from the NS/AO + PEG group ($p < 0.05$, N = 3).

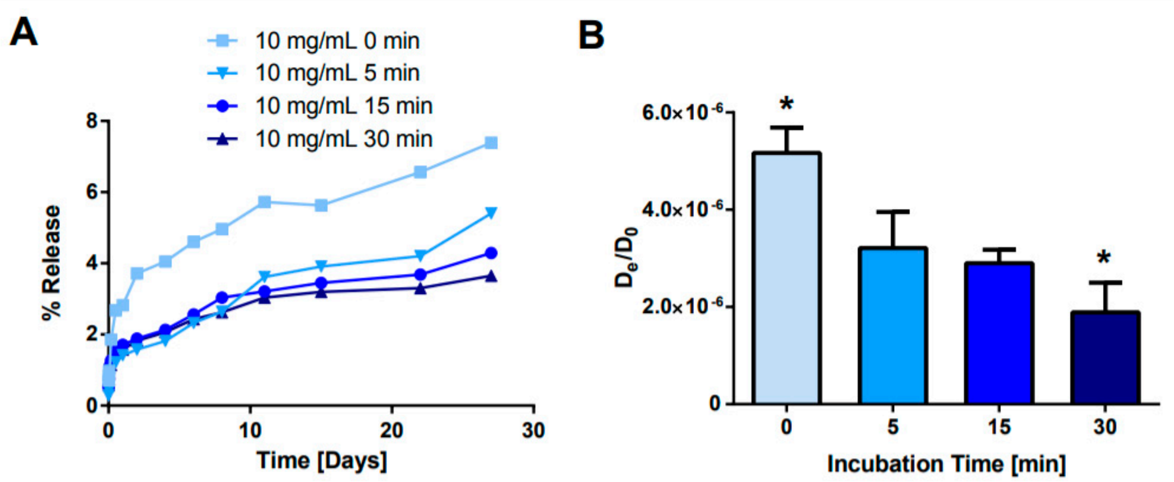


**Supplemental Figure S7**: **Influence of NS/AO Incubation Time with 10 mg/mL NS. A.** Release profiles of AO from PEG-NS hydrogels with 10 mg/mL NS and varying incubation time between NS and AO prior to addition of PEG. **B.** Calculated diffusion coefficient obtained from release profiles using **Eq. 13**. * indicates statistically significant difference from all other groups ($p < 0.05$, N = 4).

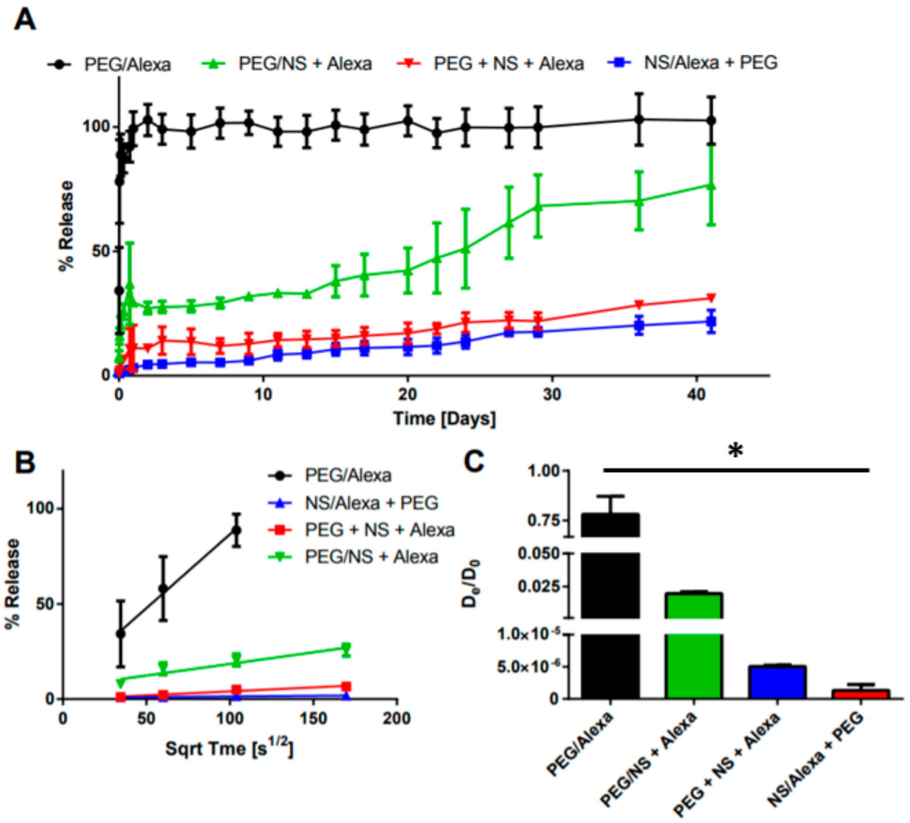


**Supplemental Figure S8: Release of Alexa 647. A.** Bulk release of Alexa from different hydrogel preparation conditions. **B.** Plot of fractional release as a function of the square root of time. **C.** Calculated effective diffusion coefficient for each condition. * indicates statistical significant difference between all groups ($p$ < 0.05, N = 4)

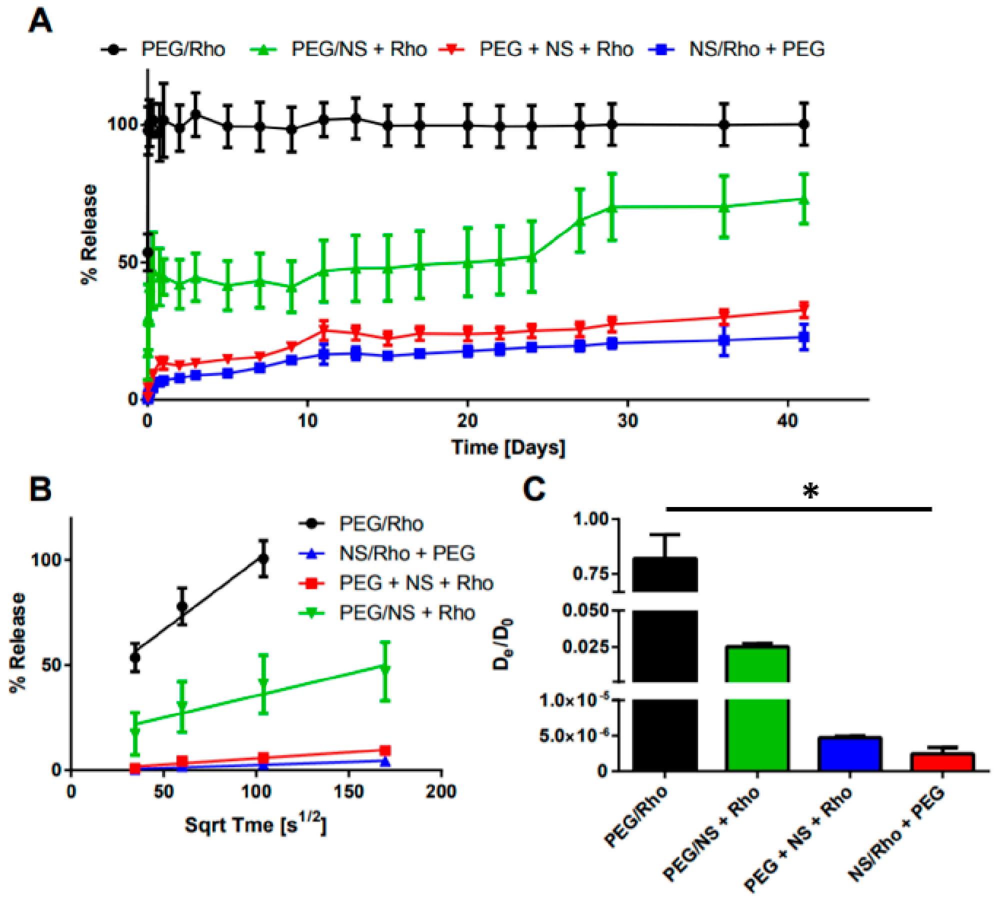


**Supplemental Figure S9: Release of Atto Rho 13. A.** Bulk release of Rho from different hydrogel preparation conditions. **B.** Plot of fractional release as a function of the square root of time. **C.** Calculated effective diffusion coefficient for each condition. * indicates statistical significant difference between all groups ($p < 0.05$, N = 4).
